# Primary healthcare workers’ COVID-19 infection status following implementation of adjusted epidemic prevention and control strategies: a cross-sectional study in Jiangsu, China

**DOI:** 10.3389/fpubh.2023.1297770

**Published:** 2023-12-22

**Authors:** Beier Lu, Rongji Ma, Jinshui Xu, Yongjie Zhang, Haijian Guo, Hualing Chen, Pengcheng Miao, Yongkang Qian, Biyun Xu, Ya Shen, Bingwei Chen

**Affiliations:** ^1^Department of Biostatistics, School of Public Health, Southeast University, Nanjing, China; ^2^Jiangsu Provincial Center for Disease Control and Prevention, Nanjing, China; ^3^Medical Statistics and Analysis Center, Nanjing Drum Tower Hospital, Nanjing University Medical School, Nanjing, China

**Keywords:** healthcare workers, COVID-19, infection, China, influencing factors

## Abstract

**Introduction:**

In times of epidemic outbreaks, healthcare workers (HCWs) emerge as a particularly vulnerable group. This cross-sectional study endeavors to assess the COVID-19 infection rate among the primary HCWs in Jiangsu Province subsequent to the implementation of adjusted epidemic prevention and control strategies.

**Methods:**

From January 17 to February 2, 2023, an extensive survey was conducted among primary HCWs in Jiangsu Province, employing a self-designed questionnaire. Logistic regression analysis was utilized to identify the factors associated with COVID-19 infection.

**Results:**

The overall infection rate among primary HCWs stood at 81.05%, with a 95% confidence interval (*CI*) of 80.61–81.48%. Among those afflicted, cough, fatigue, and fever emerged as the three most prevalent symptoms, each with an incidence rate exceeding 80%. In the context of multivariate logistic regression, an elevated risk of COVID-19 infection was observed in correlation with female gender (adjusted odds ratio [a*OR*] = 1.12, 95% CI: 1.04–1.21), possessing a bachelor’s degree or higher (a*OR* = 1.32, 95% CI: 1.23–1.41), accumulating over 10 years of work experience (a*OR* = 1.28, 95% CI: 1.11–1.47), holding a middle-level cadre position (a*OR* = 1.22, 95% CI: 1.11–1.35), assuming the role of a unit leader (a*OR* = 1.30, 95% CI: 1.11–1.54), and working in a fever clinic for 1 to 10 days per month (a*OR* = 1.42, 95% CI: 1.29–1.57). Conversely, advanced age (a*OR* = 0.76, 95% CI: 0.70–0.82), being underweight (a*OR* = 0.78, 95% CI: 0.69–0.90), current smoking (a*OR* = 0.64, 95% CI: 0.57–0.71), receiving 4 doses of COVID-19 vaccine (a*OR* = 0.49, 95% CI: 0.37–0.66), and pregnancy or perinatal status (a*OR* = 0.85, 95% CI: 0.72–0.99) were associated with a diminished risk of infection.

**Conclusion:**

Following the implementation of adjusted policies, a substantial proportion of primary HCWs in Jiangsu province contracted COVID-19. Female gender and younger age emerged as risk factors for COVID-19 infection, while no discernible link was established between professions and COVID-19 susceptibility. The receipt of COVID-19 vaccines demonstrated efficacy in curtailing the infection rate, underscoring the significance of bolstering prevention knowledge and heightening self-protective awareness among primary HCWs.

## 1 Introduction

Coronavirus disease 2019 (COVID-19), induced by severe acute respiratory syndrome coronavirus 2 (SARS-CoV-2), was initially identified among hospitalized patients in Wuhan, China, spanning December 2019 and January 2020 (1, 2). This viral pathogen underpins a potentially fatal illness, sparking profound global public health apprehensions. As of May 10, 2023, the World Health Organization’s records indicate a worldwide tally of over 765 million confirmed COVID-19 cases, encompassing in excess of 99 million cases documented in China (3).

In contrast to the general population, healthcare workers (HCWs) encounter a heightened risk of infection due to their close and direct exposure to individuals afflicted with COVID-19 (4–6). Particularly noteworthy, factors such as older age, male gender, Black individuals, Asian individuals, and minority ethnic groups, alongside underlying health conditions, contribute to an escalated risk of mortality within the HCW demographic (7–9). American participants who self-reported as African individuals and Latino participants are more likely to be at increased risk of infection and contribute to racial disparities in mortality due to living in neighborhoods with poor air quality (10), working in jobs that do not allow for telecommuting (9), or lack of access to medical care (11). As of August 10, 2021, among the 25 cross-sectional studies assessing COVID-19 prevalence, the combined prevalence of COVID-19 among HCWs, as determined through antibody tests, was found to be 7% (with 95% confidence intervals [CI]: 3–17%), in contrast to the prevalence of 11% (95% CI, 7–16%) observed in studies utilizing PCR tests (12). Among HCWs, instances of infection were predominantly observed in women and nurses, while a substantial proportion of fatalities were recorded among men and doctors (13).

On November 11 and December 7, 2022, the State Council’s Joint Prevention and Control Mechanism responded to the COVID-19 epidemic by introducing the “Twenty Measures” (14) and “Ten New Rules” (15). Notably, the “Ten New Rules” emphasize that nucleic acid testing for all staff should no longer be organized by administrative regions, thereby reducing both the extent and frequency of such testing. Additionally, the requirement for presenting evidence of negative nucleic acid results has been eliminated, except in cases involving specific institutions.

After the implementation of adjusted epidemic prevention and control strategies, the reported incidence of COVID-19 infection in China exhibited a noticeable upward trajectory, exerting a substantial impact on medical institutions across the nation. Nevertheless, primary HCWs persevered in their duties despite their own infections. To gain a comprehensive understanding of the situation surrounding primary HCWs infected with COVID-19 under the new prevention and control strategies, a survey was conducted through the basic public health service network of Jiangsu Province. The goal was to assess the prevalence and pinpoint the factors linked to COVID-19 infection among primary HCWs within Jiangsu Province amid the COVID-19 epidemic.

## 2 Materials and methods

### 2.1 Study subjects

In this cross-sectional study, the link to the questionnaire was disseminated to various categories of HCWs, including doctors, nurses, medical technicians, pharmacists, administrators, and other personnel, employed in primary healthcare institutions across the province. This distribution was facilitated through the basic public health service work network of Jiangsu Province on January 17, 2023. The IP address of each respondent during submission was recorded.

The research protocol obtained approval from the Ethics Committee of the Jiangsu Provincial Center for Disease Prevention and Control (JSJK2023-B010-01). Additionally, all participants volunteered for this study, and they signed the informed consent form at the outset of the questionnaire.

### 2.2 Definitions

After the implementation of adjusted COVID-19 prevention and control strategies, China has discontinued the universal requirement for nucleic acid testing in all individuals. In the context of this study, the term “infected” was delineated to encompass individuals meeting any of the following criteria: (1) testing positive for viral nucleic acid, (2) testing positive for antigens, (3) testing positive for both viral nucleic acid and antigens, or (4) displaying symptoms associated with COVID-19 but not undergoing nucleic acid or antigen testing. Furthermore, the term “uninfected” encompassed individuals meeting either of the following criteria: (1) displaying no positive outcomes in both viral nucleic acid testing and/or antigen testing, or (2) exhibiting no symptoms linked to COVID-19 and refraining from nucleic acid or antigen testing.

The body mass index (BMI) was calculated by dividing the weight in kilograms by the square of height in meters. The BMI classification criteria for Chinese adults are outlined as follows (16): underweight is defined as BMI < 18.5 kg/m^2^, normal weight is defined as BMI 18.5–23.9 kg/m^2^, overweight is defined as BMI 24.0–27.9 kg/m^2^, and obesity was defined as BMI ≥ 28.0 kg/m^2^.

### 2.3 Survey measures

The questionnaire used in this study was adapted from the second round of COVID-19 Infection Questionnaire released by China’s National Center for Disease Control and Prevention and Peking Union Medical College, which includes 45-items. In accordance with the Program for Prevention and Control of COVID-19 Infections (10th Edition, http://www.nhc.gov.cn/ylyjs/pqt/202301/32de5b2ff9bf4eaa88e75bdf7223a65a.shtml), the final questionnaire was confirmed in two rounds of discussions with five epidemiologists and statistical experts. The questionnaire consists of 78 obligatory questions, ensuring data integrity in this study. It comprises four sections: (1) Basic information: this includes demographic characteristics, past medical history, and lifestyle, (2) Vaccination status: this encompasses information about the doses and types of COVID-19 vaccine, influenza vaccine, pneumonia vaccine, (3) Clinical manifestations and COVID-19 outcomes: this section covers over ten symptoms such as fever, muscle aches, and cough, along with the duration of symptoms, hospital visits, and medications taken, and (4) Work burden: this includes factors like years of work experience, daily working hours, work-related stress, and whether individuals worked in a fever clinic.

Questionnaire completion and data collection were carried out based on the China’s largest online survey platform (Questionnaire Star, https://www.wjx.cn). Questionnaire Star is a professional service platform for electronic questionnaire design and data collection, which has been widely used by researchers. Designated personnel managed the export of the database through project-specific accounts.

### 2.4 Statistical analysis

In accordance with prior research (17), the COVID-19 infection rate of the faculty and students at the School of Public Health in universities in Beijing, subsequent to the implementation of adjusted epidemic prevention and control strategies, was 77.89% before December 23, 2022. Assuming a prevalence of COVID-19 of 80%, a significance level (*α*) of 0.05, and a relative error of sampling of 3%, we estimated the sample size of each city to be 1,107 individuals, and there were 13 cities in the whole province, and considering the design effect of 2, the sample size was 28,782 individuals.

Continuous variables were expressed as means (standard deviation, SD), while categorical variables were presented as frequencies and percentages. Statistical differences between groups were assessed using two sample t-test for continuous variables and Pearson’s Chi-square test or Fisher’s exact test for categorical variables. To identify factors associated with COVID-19 infection among Primary HCWs, both univariate and multivariate logistic regression analyzes were conducted. Variables associated with infection at *p* ≤ 0.1 in the univariate analysis were included in the multivariate logistic stepwise regression analysis. The risk of COVID-19 infection was assessed with odds ratios (*OR*) and 95% *CI*, or adjusted odds ratios (a*OR*) and 95% CI. All statistical analyzes were performed using R software (R version 4.3.0). A two-sided *p* ≤ 0.05 was considered statistically significant.

## 3 Results

### 3.1 Participants

The average number of primary HCWs per primary healthcare institution in Jiangsu Province is 104. We randomly sampled 400 primary healthcare institutions and collected a total of 34,090 questionnaires between January 17 and February 2, with a roughly estimated response rate of 81.95%. After excluding questionnaires with logical errors or those completed in an unreasonable time frame (less than 300 s for infected individuals and less than 210 s for uninfected individuals), we ultimately acquired 31,482 valid questionnaires, constituting 92.3% of the total number of questionnaires.

### 3.2 Demographic characteristics of primary HCWs

In this study, all results are analyzed based on the participation of 31,482 primary HCWs. The mean age was 39.62 years, with 67% falling within the age range of 45 years or younger, while the remaining 33% were older than 45 years. Of the participants, 9,439 (30%) were men and 22,043 (70%) were women. In total, 17,393 (55%) held a bachelor’s degree or higher, and the majority of participants were doctors (46%) and nurses (30%). For a comprehensive breakdown of the participants’ demographic characteristics, please refer to Table 1.

**Table 1:** Demographic characteristics of primary HCWs in Jiangsu Province.

Variable		Total, *N* (%)
Gender	Male	9,439 (30%)
	Female	22,043 (70%)
Age (years)	Mean (SD)	39.62 (10.72)
	≤45	20,965 (67%)
	>45	10,517 (33%)
BMI	Underweight	1,556 (5%)
	Normal weight	16,806 (54%)
	Overweight	9,546 (30%)
	Obesity	3,574 (11%)
Profession	Doctor	14,657 (46%)
	Nurse	9,319 (30%)
	Medical technician	2,856 (9%)
	Pharmacist	2,092 (7%)
	Administrator	645 (2%)
	Other personnel	1,913 (6%)
Position	Staff	26,703 (85%)
	Middle-level cadre	3,728 (12%)
	Unit leader	1,051 (3%)
Education level	Below a bachelor’s degree	14,089 (45%)
	A bachelor’s degree or higher	17,393 (55%)
Work experience (years)	0–1	1,500 (5%)
	2–4	3,108 (10%)
	5–10	5,700 (18%)
	>10	21,174 (67%)

### 3.3 The prevalence and determinants of COVID-19 infection

Following the implementation of adjusted epidemic prevention and control strategies, out of the 31,482 valid questionnaires, 25,516 respondents were infected with COVID-19 and 5,966 remained uninfected, yielding an infection rate of 81.05% (95% CI: 80.61–81.48%).

The in-depth analysis of the factors contributing to COVID-19 infection among the primary HCWs are presented in Table 2. The findings indicated that females exhibited a higher infection rate (83%) in comparison to males (77%). In the younger age group (≤ 45 years old), 83% of individuals were infected, while the infection rate among those over 45 years old was 76%. The prevalence of infection was more pronounced among participants with a bachelor’s degree or higher (84%) than those with below a bachelor’s degree (77%). Among all professional roles, nurses displayed the highest infection rate at 84%, while doctors exhibited the lowest rate at 78%. For primary HCWs who received 0, 1, 2, 3, and 4 doses of the COVID-19 vaccine, the corresponding infection rates were 87, 87, 85, 84, and 76%, with statistically significant differences observed between the infected and uninfected groups in terms of gender, age, education level, profession, and COVID-19 vaccine doses (*p* < 0.001).

**Table 2:** Univariate analysis for factors associated with COVID-19 infection.

Variable	Uninfected *n* = 5,966	Infected *n* = 25,516	Total	*p*-value	OR (95% CI)
*Gender*				<0.001	
Male	2,217 (23%)	7,222 (77%)	9,439		Ref
Female	3,749 (17%)	18,294 (83%)	22,043		**1.50 (1.41–1.59)**
*Age (years)*				<0.001	
Mean (SD)	41.37 (11.68)	39.21 (10.44)			
≤45	3,467 (17%)	17,498 (83%)	20,965		Ref
>45	2,499 (24%)	8,018 (76%)	10,517		**0.64 (0.60–0.67)**
*BMI*				<0.001	
Normal weight	2,976 (18%)	13,830 (82%)	16,806		Ref
Underweight	315 (20%)	1,241 (80%)	1,556		**0.85 (0.74–0.97)**
Overweight	1,947 (20%)	7,599 (80%)	9,546		**0.84 (0.79–0.90)**
Obesity	728 (20%)	2,846 (80%)	3,574		**0.84 (0.77–0.92)**
*Education level*				<0.001	
Below a bachelor’s degree	3,232 (23%)	10,857 (77%)	14,089		Ref
A bachelor’s degree or higher	2,734 (16%)	14,659 (84%)	17,393		**1.60 (1.51–1.69)**
*Work experience (years)*				<0.001	
0–1	309 (21%)	1,191 (79%)	1,500		Ref
2–4	560 (18%)	2,548 (82%)	3,108		**1.18 (1.01–1.38)**
5–10	948 (17%)	4,752 (83%)	5,700		**1.30 (1.13–1.50)**
>10	4,149 (20%)	17,025 (80%)	21,174		1.06 (0.94–1.21)
*Profession*				<0.001	
Doctor	3,157 (22%)	11,500 (78%)	14,657		Ref
Nurse	1,480 (16%)	7,839 (84%)	9,319		**1.45 (1.36–1.56)**
Medical technician	499 (17%)	2,357 (83%)	2,856		**1.30 (1.17–1.44)**
Pharmacist	365 (17%)	1,727 (83%)	2092		**1.30 (1.15–1.46)**
Administrator	113 (18%)	532 (82%)	645		**1.29 (1.05–1.59)**
Other personnel	352 (18%)	1,561 (82%)	1,913		**1.22 (1.08–1.38)**
*Position*				<0.001	
Staff	5,142 (19%)	21,561 (81%)	26,703		Ref
Middle-level cadre	619 (17%)	3,109 (83%)	3,728		**1.20 (1.09–1.31)**
Unit leader	205 (20%)	846 (80%)	1,051		0.98 (0.84–1.15)
*Smoking status*				<0.001	
Never-smoker	4,894 (18%)	22,741 (82%)	27,635		Ref
Former smoker	213 (23%)	696 (77%)	909		**0.70 (0.60–0.82)**
Current smoker	859 (29%)	2,079 (71%)	2,938		**0.52 (0.48–0.57)**
*Drinking status*				<0.001	
Never-drinker	4,518 (18%)	20,573 (82%)	25,091		Ref
Former drinker	378 (22%)	1,364 (78%)	1,742		**0.79 (0.70–0.89)**
Current drinker	1,070 (23%)	3,579 (77%)	4,649		**0.73 (0.68–0.79)**
*Dietary structure*				0.24	
More meat and less vegetables	733 (18%)	3,314 (82%)	4,047		Ref
Balance of meat and vegetables	4,331 (19%)	18,262 (81%)	22,593		0.93 (0.86–1.02)
Less meat and more vegetables	902 (19%)	3,940 (81%)	4,842		0.97 (0.87–1.08)
*COVID-19 vaccine doses*				<0.001	
0	55 (13%)	354 (87%)	409		Ref
1	39 (13%)	257 (87%)	296		1.02 (0.66–1.59)
2	226 (15%)	1,267 (85%)	1,493		0.87 (0.63–1.20)
3	2,615 (16%)	14,225 (84%)	16,840		0.85 (0.63–1.13)
4	3,031 (24%)	9,413 (76%)	12,444		**0.48 (0.36–0.64)**
*Influenza vaccination*				0.014	
No	4,500 (19%)	19,693 (81%)	24,193		Ref
Yes	971 (20%)	3,817 (80%)	4,788		**0.90(0.83–0.97)**
Can not remember	495 (20%)	2,006 (80%)	2,501		0.93 (0.83–1.03)
*Pneumonia vaccination*				0.004	
No	5,796 (19%)	24,950 (81%)	30,746		Ref
Yes	170 (23%)	566 (77%)	736		**0.77 (0.65–0.92)**
*Tumor*				0.69	
No	5,860 (19%)	25,043 (81%)	30,903		Ref
Yes	106 (18%)	473 (82%)	579		1.04 (0.84–1.29)
*Allergic disease*				0.91	
No	5,582 (19%)	23,864 (81%)	29,446		Ref
Yes	384 (19%)	1,652 (81%)	2,036		1.01 (0.90–1.13)
*Hemodialysis*				>0.99	
No	5,964 (19%)	25,508 (81%)	31,472		Ref
Yes	2 (20%)	8 (80%)	10		0.94 (0.20–4.41)
*Diabetes*				<0.001	
No	5,761 (19%)	24,893 (81%)	30,654		Ref
Yes	205 (25%)	623 (75%)	828		**0.70 (0.54–0.74)**
*Cardio-cerebrovascular disease*				<0.001	
No	5,443 (19%)	23,682 (81%)	29,125		Ref
Yes	523 (22%)	1,834 (78%)	2,357		**0.81 (0.73–0.89)**
*Chronic pulmonary disease*				0.057	
No	5,918 (19%)	25,366 (81%)	31,284		Ref
Yes	48 (24%)	150 (76%)	198		0.73 (0.53–1.01)
*Chronic liver disease*				0.052	
No	5,923 (19%)	25,385 (81%)	31,308		Ref
Yes	43 (25%)	131 (75%)	174		0.71 (0.50–1.00)
*Chronic kidney disease*				0.46	
No	5,948 (19%)	25,423 (81%)	31,371		Ref
Yes	18 (16%)	93 (84%)	111		1.21 (0.73–2.00)
*Immunodeficiency*				0.32	
No	5,926 (19%)	25,313 (81%)	31,239		Ref
Yes	40 (16%)	203 (84%)	243		1.19 (0.85–1.67)
*Mental illness*				0.98	
No	5,953 (19%)	25,460 (81%)	31,413		Ref
Yes	13 (19%)	56 (81%)	69		1.01 (0.55–1.84)
*Pregnancy or perinatal status*				0.022	
No	5,775 (19%)	24,540 (81%)	30,315		Ref
Yes	191 (16%)	976 (84%)	1,167		**1.20 (1.03–1.41)**
*Working in a fever clinic*				0.028	
No	3,182 (19%)	13,206 (81%)	16,388		Ref
Yes	2,784 (18%)	12,310 (82%)	15,094		**1.07 (1.01–1.13)**
*Duration of days working in the fever clinic per month (days)*				<0.001	
0	3,182 (19%)	13,206 (81%)	16,388		Ref
1–10	591 (14%)	3,684 (86%)	4,275		**1.50 (1.37–1.65)**
11–20	271 (17%)	1,315 (83%)	1,586		**1.17 (1.02–1.34)**
>20	1,922 (21%)	7,311 (79%)	9,233		**0.92 (0.86–0.98)**
*Daily working hours*				<0.001	
≤4	82 (19%)	350 (81%)	432		Ref
5–8	3,117 (17%)	15,076 (83%)	18,193		1.13 (0.89–1.45)
9–12	2,372 (21%)	8,718 (79%)	11,090		0.86 (0.67–1.10)
>12	395 (22%)	1,372 (78%)	1,767		0.81 (0.62–1.06)
*Work-related stress*				0.62	
Low	259(18%)	1,168 (82%)	1,427		Ref
Moderate	2,722 (19%)	11,707 (81%)	14,429		0.95 (0.83–1.10)
High	2,218 (19%)	9,287 (81%)	11,505		0.93 (0.81–1.07)
Extreme	767 (19%)	3,354 (81%)	4,121		0.97 (0.83–1.13)

Data are mean (SD), *n* (%). *p* values were calculated by *t* test, *χ*^2^ test, or Fisher’s exact test, as appropriate. OR, Odds ratio; CI, confidence interval. Bold value indicates that OR values are statistically significant.

No statistically significant differences were observed between the infected and uninfected groups in terms of dietary structure (*p* = 0.24), tumor (*p* = 0.69), allergic diseases (*p* = 0.91), hemodialysis (*p* > 0.99), chronic kidney disease (*p* = 0.46), immunodeficiency (*p* = 0.32), mental illness (*p* = 0.98), and work-related stress (*p* = 0.62). The individual characteristics of both the infected and uninfected groups are presented in Table 2.

### 3.4 Assessment of factors associated with COVID-19 infection among primary HCWs

The outcomes of multivariate logistic regression analysis are displayed in Table 3. The analysis revealed that an escalated risk of COVID-19 infection was correlated with female gender (a*OR* = 1.12, 95% CI: 1.04–1.21), possessing a bachelor’s degree or higher (a*OR* = 1.32, 95% CI: 1.23–1.41), accumulating over 10 years of work experience (a*OR* = 1.28, 95% CI: 1.11–1.47), holding a middle-level cadre position (a*OR* = 1.22, 95% CI: 1.11–1.35), assuming the role of a unit leader (a*OR* = 1.30, 95% CI: 1.11–1.54), and working in a fever clinic for 1 to 10 days per month (a*OR* = 1.42, 95% CI: 1.29–1.57). Conversely, advanced age (a*OR* = 0.76, 95% CI: 0.70–0.82), being underweight (a*OR* = 0.78, 95% CI: 0.69–0.90), current smoking (a*OR* = 0.64, 95% CI: 0.57–0.71), receiving 4 doses of COVID-19 vaccine (a*OR* = 0.49, 95% CI: 0.37–0.66), and pregnancy or perinatal status (a*OR* = 0.85, 95% CI: 0.72–0.99) were linked to a diminished risk of infection.

**Table 3:** Factors associated with COVID-19 infection in multivariate logistic regression.

Variables	aOR (95% CI)	*p*
Gender		
Male	Ref	
Female	**1.12 (1.04–1.21)**	0.005
Age (years)		
≤45	Ref	
>45	**0.76 (0.70–0.82)**	<0.001
BMI		
Normal weight	Ref	
Underweight	**0.78 (0.69–0.90)**	<0.001
Overweight	1.00 (0.93–1.06)	0.887
Obesity	0.99 (0.90–1.08)	0.784
Education level		
Below a bachelor’s degree	Ref	
A bachelor’s degree or higher	**1.32 (1.23–1.41)**	<0.001
Work experience (years)		
0–1	Ref	
2–4	1.11 (0.94–1.30)	0.210
5–10	1.11 (0.95–1.29)	0.188
>10	**1.28 (1.11–1.47)**	<0.001
Position		
Staff	Ref	
Middle-level cadre	**1.22 (1.11–1.35)**	<0.001
Unit leader	**1.30 (1.11–1.54)**	0.001
Smoking status		
Never-smoker	Ref	
Former smoker	0.90 (0.76–1.06)	0.210
Current smoker	**0.64 (0.57–0.71)**	<0.001
COVID-19 vaccine doses		
0	Ref	
1	0.99 (0.63–1.54)	0.963
2	0.84 (0.61–1.15)	0.278
3	0.87 (0.65–1.16)	0.348
4	**0.49 (0.37–0.66)**	<0.001
Pneumonia vaccination		
Yes	0.86 (0.72–1.03)	0.098
Pregnancy or perinatal status		
Yes	**0.85 (0.72–0.99)**	0.047
Duration of days working in the fever clinic per month (days)		
0	Ref	
1–10	**1.42 (1.29–1.57)**	<0.001
11–20	1.13 (0.99–1.30)	0.079
>20	1.00 (0.94–1.07)	0.958
Daily working hours		
≤4	Ref	
5–8	1.22 (0.95–1.56)	0.122
9–12	1.02 (0.80–1.32)	0.859
>12	1.01 (0.77–1.33)	0.931

aOR, adjusted odds ratio; CI, confident interval. Bold value indicates that OR values are statistically significant.

### 3.5 Symptoms observed in infected patients

**Figure 1:**
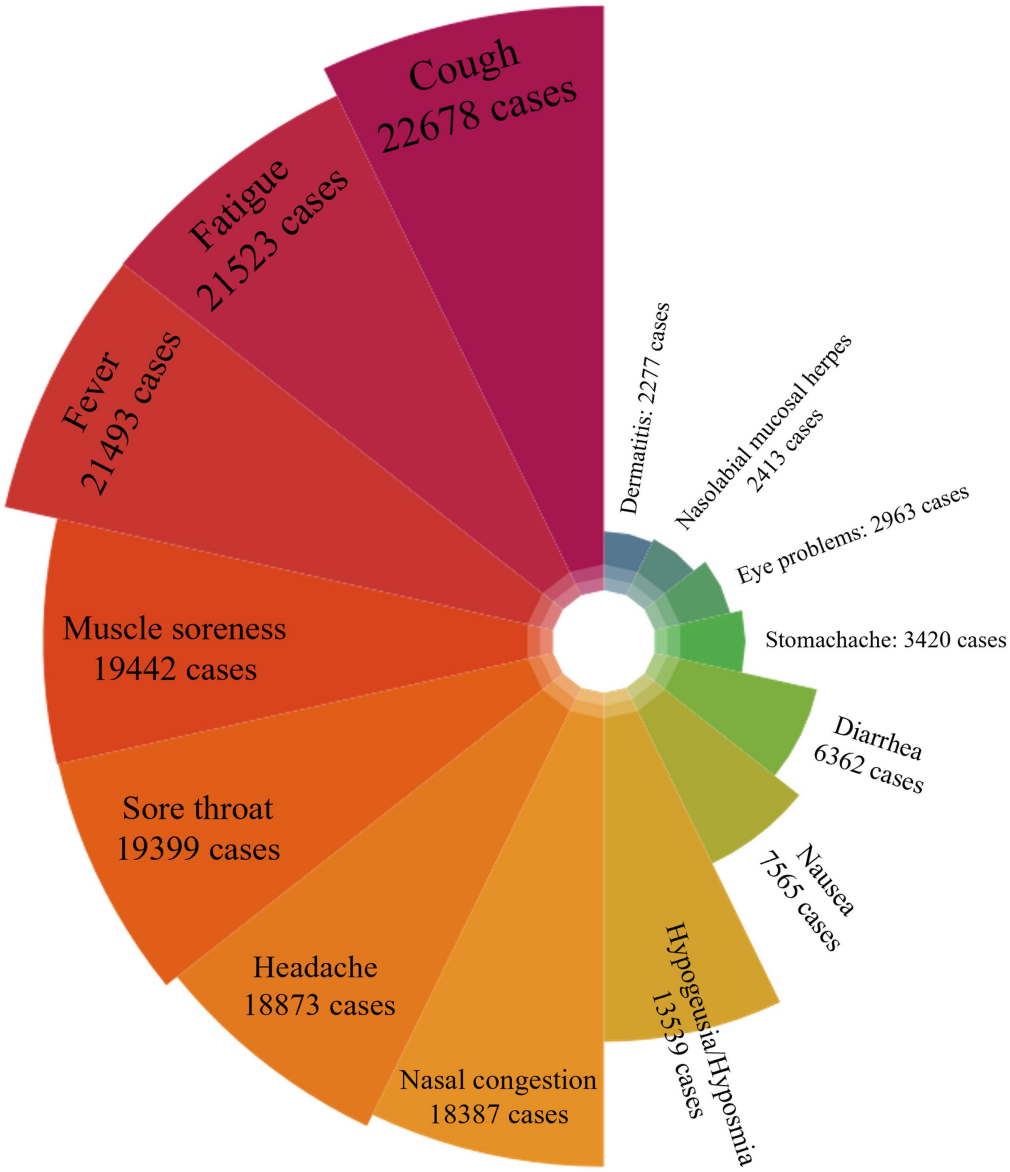
The nightingale rose diagram: illustration of the symptoms observed in infected patients.

The symptoms observed in infected patients are presented in Figure 1. As per the survey, cough emerged as the most prevalent symptom among the infected patients, being reported by approximately 89% (22,678/25,516) of them. Following this, other frequently encountered symptoms included fatigue (21,523 [84%]) and fever (21,493 [84%]). For a more detailed account of the symptoms, please refer to the Supplementary material provided in an additional work document.

## 4 Discussion

This study offers the initial insight into the infection status of primary HCWs in Jiangsu Province, China, during the first wave of the COVID-19 epidemic, following the implementation of adjusted policies. By the conclusion of the survey, a mere 57 days after the issuance of the “Ten New Rules,” the aggregate infection rate among primary HCWs in Jiangsu province had surged to 81.05%. Li et al. conducted a comparable study and highlighted that COVID-19 infection in China was spreading at an accelerated pace compared to previous years, leading to a rapid escalation in infection rates among Chinese residents (18). The impending arrival of the Spring Festival (January 21) led to a notable surge in population movement, as individuals returned to their hometowns ahead of our survey commencement. This influx of travelers, including those who tested positive for viral nucleic acid or antigens, potentially played a role in the observed high infection rate.

In this study, the infection rate was higher among females (83%) in comparison to males (77%). Multivariate logistic regression analysis revealed that female gender constituted a risk factor for COVID-19 infection (a*OR* = 1.12). Existing research has consistently indicated that gender significantly influences the risk of COVID-19 infection across diverse workplaces. This trend is particularly noticeable in care-related occupations, where women tend to face elevated susceptibility to the risk of COVID-19 infection (19). In this outbreak, some primary HCWs will be dispatched from primary healthcare institutions in the province to conduct household follow-up visits to key populations in order to keep abreast of the development of the condition of positive people. This team is mainly undertaken by nursing staff, most of whom are young females, thus they indirectly increasing the chances of contact with exposures in society and have a higher risk of contracting COVID-19. In addition, women have more chances to come into contact with social infectious agents outside of working hours, such as shopping for groceries, going to the supermarket to buy household goods, and picking up and dropping off their children at school.

Notably, younger primary HCWs exhibited a higher likelihood of COVID-19 infection. Multivariate regression analysis unveiled an *OR* of 0.76 for individuals over 45 years old, in contrast to those aged 45 years or younger. This finding is in line with the research conducted by scientists in Madagascar (20). However, previous studies have consistently indicated that the risk of COVID-19 infection escalates significantly with advanced age (9, 21, 22). Research has demonstrated that advanced age independently correlates with a greater viral load, potentially linked to the decline of immune function among the older adult and the manifestation of less conspicuous symptoms (23). Another plausible interpretation for the outcomes of this study could be that younger individuals may have lowered their guard and exercised fewer precautions in their professional and daily lives due to an inadequate awareness of self-protection. Additionally, it is plausible that, drawing from prior studies (9, 21, 22), hospital administrators may have intentionally allocated tasks to older workers that did not require close contact with COVID-19 patients, or alternatively, permitted them to work remotely as a measure to minimize exposure (24). Further, the accelerated development of herd immunity within the younger population might potentially result in fewer severe cases (25).

Due to the high incidence of infections among young people, this may cause a significant short-term shock to the healthcare service, leading to greater strain on the healthcare system. Although there were differences in infection rates between men and women, both groups exhibited a higher infection rate. Therefore, before the next outbreak, it is necessary to have a back-up population and to adopt a shift system for primary HCWs, which may avoid a shortage of critical care staff or a breakdown of the entire healthcare system.

Upon adjusting for age, gender, and other confounding factors, we observed a notable decrease in the relative risk of COVID-19 infection associated with the administration of a fourth dose of COVID-19 vaccine. In comparison to individuals who had never received the COVID-19 vaccine, those who had received four doses exhibited an *OR* of 0.49. From December 1, 2022 to February 6, 2023, the prevalent strains of the new coronavirus in China were BA.5.2.48 and BF.7.14, both subbranches of the Omicron variant BA.5 (26). Researches have established that a two-dose regimen of BNT162b2 conferred 95% protection against COVID-19 in persons 16 years of age or older (27), a third dose of the COVID-19 vaccine offers effective protection against Omicron (28, 29), although these protection wane over time, a fourth dose has the potential to restore antibody levels (30). BNT162b2 vaccine has been shown to be highly effective in preventing COVID-19 symptomatic infections, as well as for the more serious outcomes: hospitalization, severe illness, and death (31). Among adults older than 55 years who had received 3 doses of BNT162b2, immunogenicity against Omicron BA.1 increased considerably with the omicron BA.1–adapted BNT162b2 vaccines than with the original dose of BNT162b2 (32). In our study, the collective infection rate among primary HCWs reached 81.05%. Conversely, among those who had received the fourth dose of the COVID-19 vaccine, the infection rate diminished to 76%. However, only 40% of primary HCWs had received the fourth dose of the vaccine, with 2% of primary HCWs either had received a single dose or remained unvaccinated. This suggested the necessity for maintaining COVID-19 vaccination, the need for prompt promotion of COVID-19 booster immunization for HCWs and gradually extension of such efforts to the entire population. This approach will not only safeguard HCWs but also curtail the risk of potential nosocomial infections among hospitalized patients.

Among all professional roles, nurses exhibited the highest infection (84%), while doctors had the lowest rate at 78%. The average age of nurses stood at 35.08 years, contrasted with the average age of non-nurse staff at 41.52 years. Given that younger age has emerged as a risk factor for COVID-19 infection in this study, the observed age discrepancy could potentially contribute to the elevated infection rate observed among nurses. Univariate analysis outcomes indicated that nurses, medical technicians, pharmacists, administrators, and others faced a heightened risk of COVID-19 infection compared to doctors. However, after accounting for confounding factors, no discernible association surfaced between professions and COVID-19 infection among primary HCWs, aligning with other studies (20, 33). While other research (5, 34–37) has demonstrated that frontline HCWs in close proximity to COVID-19 patients encounter significantly greater infection risks, our study did not yield this evidence. This could be attributed to the release of the “Ten New Rules” in China, which eliminated the requirement for centralized isolation for positive patients, opting for home isolation whenever possible. Moreover, mandatory nucleic acid testing was abolished. Consequently, a significant number of asymptomatic cases are present, escalating the risk of exposure in the absence of proper personal protective equipment (PPE) (38, 39). It has been confirmed that exposure to COVID-19-positive family members or co-workers considerably amplifies the risk of COVID-19 infection (33, 40). Although hospitals make it mandatory for primary HCWs to wear PPE, which is impossible to be worn at all times, so primary HCWs can be exposed in the community or infected at home. Collective transmission within households might be a primary factor fueling the rapid dissemination of this epidemic.

This study possessed several strengths: the respondents were HCWs in primary healthcare institutions, ensuring easy access to samples that were highly cooperative. Moreover, the majority of respondents possessed both preventive and medical knowledge, enhancing the accuracy of self-reported results. The quality of response data demonstrated a high level of accuracy, contributing to the reliability of the findings. Additionally, the study indirectly provided insight into the infection rate of a specific group.

However, our study did entail certain limitations. Firstly, given that nucleic acid testing is no longer obligatory, the group of individuals who displayed no COVID-19-related symptoms and did not undergo nucleic acid or antigen testing might encompass asymptomatic cases. Consequently, the self-reported overall infection rate among the subjects examined in this study might potentially underestimate the actual infection rate. Secondly, our questionnaire was not designed to be comprehensive enough, for example, it lacked information on travel modes, compliance with precautionary measures, and the extent of PPE utilization, all of which could constitute significant factors influencing virus transmission. Thirdly, as a cross-sectional survey, our study’s scope was limited to a snapshot in time, preventing the capture of the dynamic progression of the epidemic, and due to the online self-administered format, we did not capture critically ill patients or those who died and older adult people may not be proficient in cell phone operation, potentially leading to selection bias. Fourthly, although a pre-survey was conducted with our team and 20 graduate students majoring in public health before the actual survey to revise the question formulation, the final questionnaire was filled out remotely and lacked on-site communication and explanation, and thus there may have been a certain bias in the understanding of the same question between the questionnaire filler and researchers.

## 5 Conclusion

Our study aimed to assess the prevalence and pinpoint the factors linked to COVID-19 infection among primary HCWs in Jiangsu Province subsequent to the implementation of adjusted epidemic prevention and control strategies. The results showed that the overall infection rate among primary HCWs stood at 81.05%, with a 95% *CI* of 80.61–81.48%. Being younger and female primary HCWs were noted risk factors for COVID-19 infection, and COVID-19 vaccines can significantly mitigate the infection rate. In conclusion, assessment of infection risks confronted by HCWs during the initial wave of the COVID-19 epidemic, after the implementation of adjusted epidemic prevention and control strategies, stands to improve preparedness for timely protective actions in anticipation of subsequent epidemic waves. This proactive approach will be instrumental in substantially curtailing the occurrence of re-positive HCWs and reducing the infection rate within the broader population.

## Data availability statement

The raw data supporting the conclusions of this article will be made available by the authors, without undue reservation.

## Ethics statement

The studies involving humans were approved by the Ethics Committee of the Jiangsu Provincial Center for Disease Prevention and Control (Reference number: JSJK2023-B010-01). The studies were conducted in accordance with the local legislation and institutional requirements. The participants provided their written informed consent to participate in this study.

## Author contributions

BL: Data curation, Formal analysis, Software, Writing – original draft, Investigation. RM: Data curation, Formal analysis, Software, Writing – original draft, Investigation. JX: Investigation, Project administration, Resources, Supervision, Writing – review & editing. YZ: Investigation, Project administration, Resources, Supervision, Writing – review & editing. HG: Funding acquisition, Investigation, Resources, Supervision, Writing – review & editing. HC: Data curation, Investigation, Software, Writing – review & editing. PM: Investigation, Software, Writing – review & editing. YQ: Investigation, Software, Writing – review & editing. BX: Funding acquisition, Investigation, Writing – review & editing. YS: Conceptualization, Writing – review & editing, Investigation, Supervision, Resources. BC: Conceptualization, Writing – review & editing, Data curation, Investigation, Supervision.
