# Supplementary material for: Primary healthcare workers’ COVID-19 infection status following implementation of adjusted epidemic prevention and control strategies: a cross-sectional study in Jiangsu, China
Source: Front Public Health. 2023 Dec 22;11:1297770. doi: 10.3389/fpubh.2023.1297770 (PMC10770863; doi:10.3389/fpubh.2023.1297770)
Supplement: Supplementary file 1 [file Table_1.DOCX]

Supplementary Material

# Supplementary Figures and Tables

Table S1. Symptoms of infected patients

| **Symptoms** | **Frequency** | **Percent (%)** |
| --- | --- | --- |
| Cough | 22678 | 89 |
| Fatigue | 21523 | 84 |
| Fever | 21493 | 84 |
| Muscle soreness | 19442 | 76 |
| Sore throat | 19399 | 76 |
| Headache | 18873 | 74 |
| Nasal congestion | 18387 | 72 |
| Hypogeusia/Hyposmia | 13539 | 53 |
| Nausea | 7565 | 30 |
| Diarrhea | 6362 | 25 |
| Stomachache | 3420 | 13 |
| Eye problems | 2963 | 12 |
| Nasolabial mucosal herpes | 2413 | 9 |
| Dermatitis | 2277 | 9 |
